# Supplementary material for: Separation and Identification of Resveratrol Butyrate Ester Complexes and Their Bioactivity in HepG2 Cell Models
Source: Int J Mol Sci. 2021 Dec 17;22(24):13539. doi: 10.3390/ijms222413539 (PMC8703675; doi:10.3390/ijms222413539)
Supplement: Supplementary file 1 [file ijms-22-13539-s001.zip › ijms-1493247-supplementary.pdf]

Supplementary Materials:

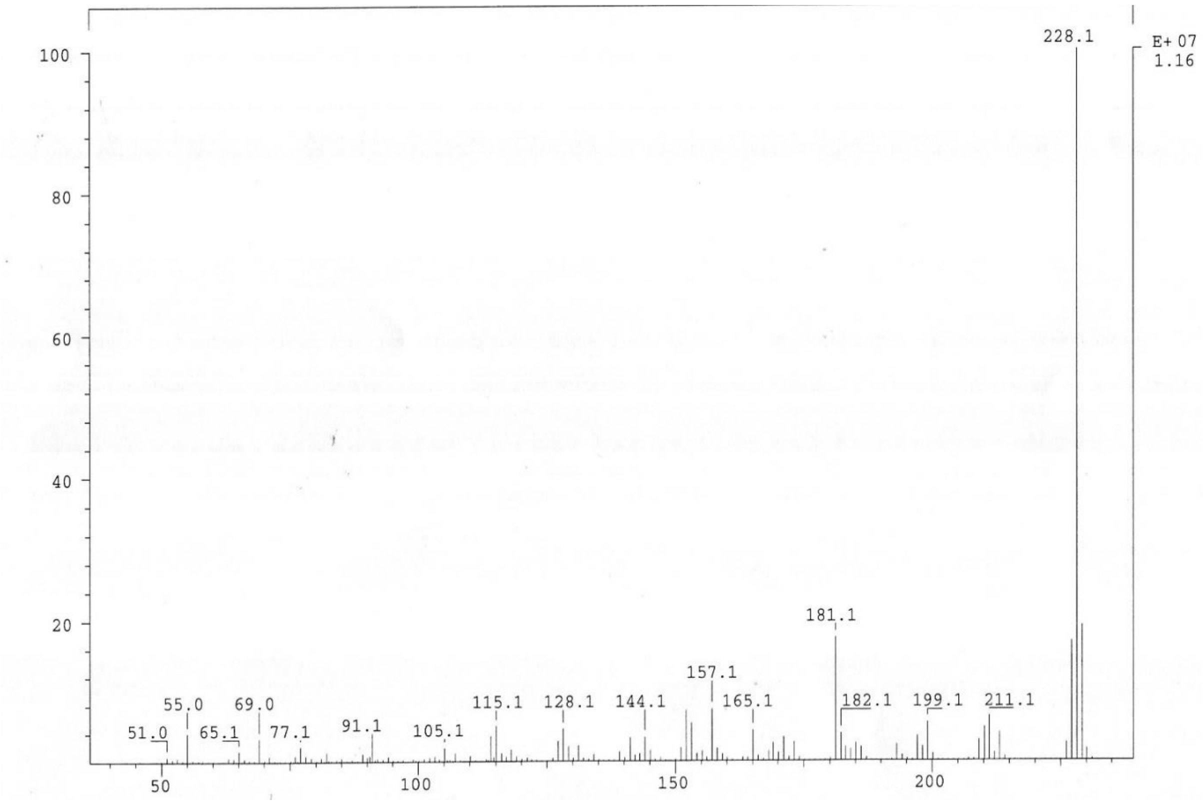

Figure S1: Mass Spectrum of compound **1** (EI-MS Spectrum)

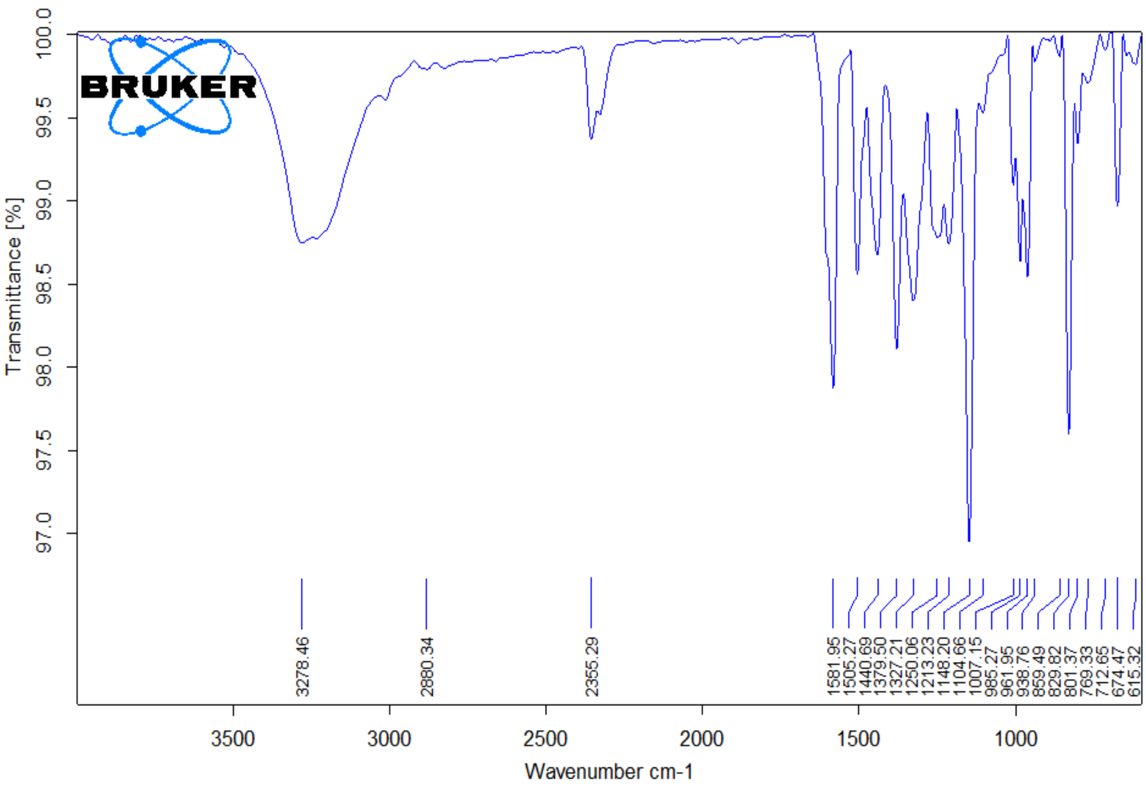

Figure S2: IR spectrum of compound **1**

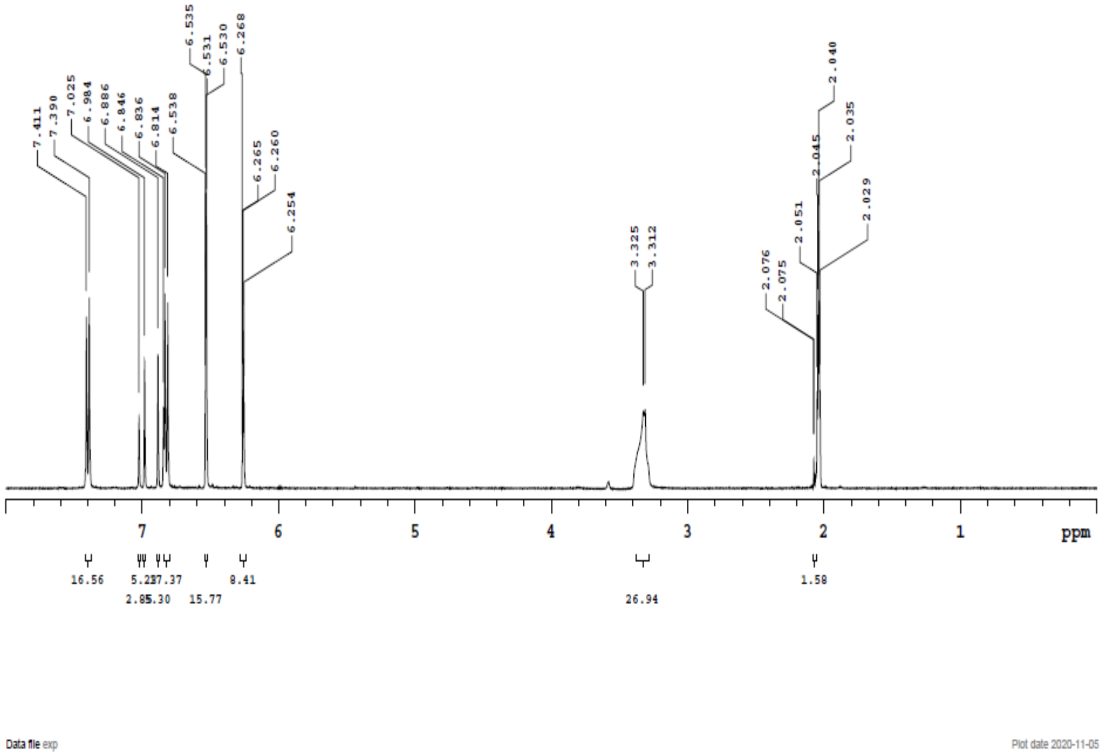

Figure S3: ^{1}H-NMR spectrum of compound **1** in CD_3COCD_3

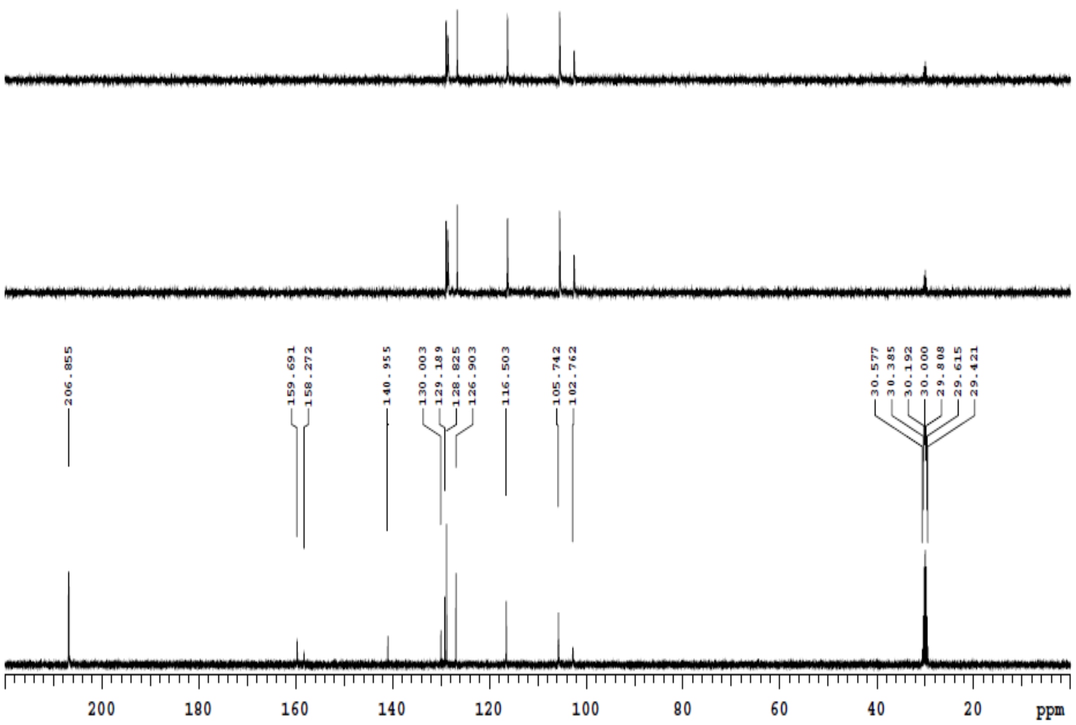

Figure S4: ^{13}C-NMR spectrum and DEPT of compound **1** in CD_3COCD_3

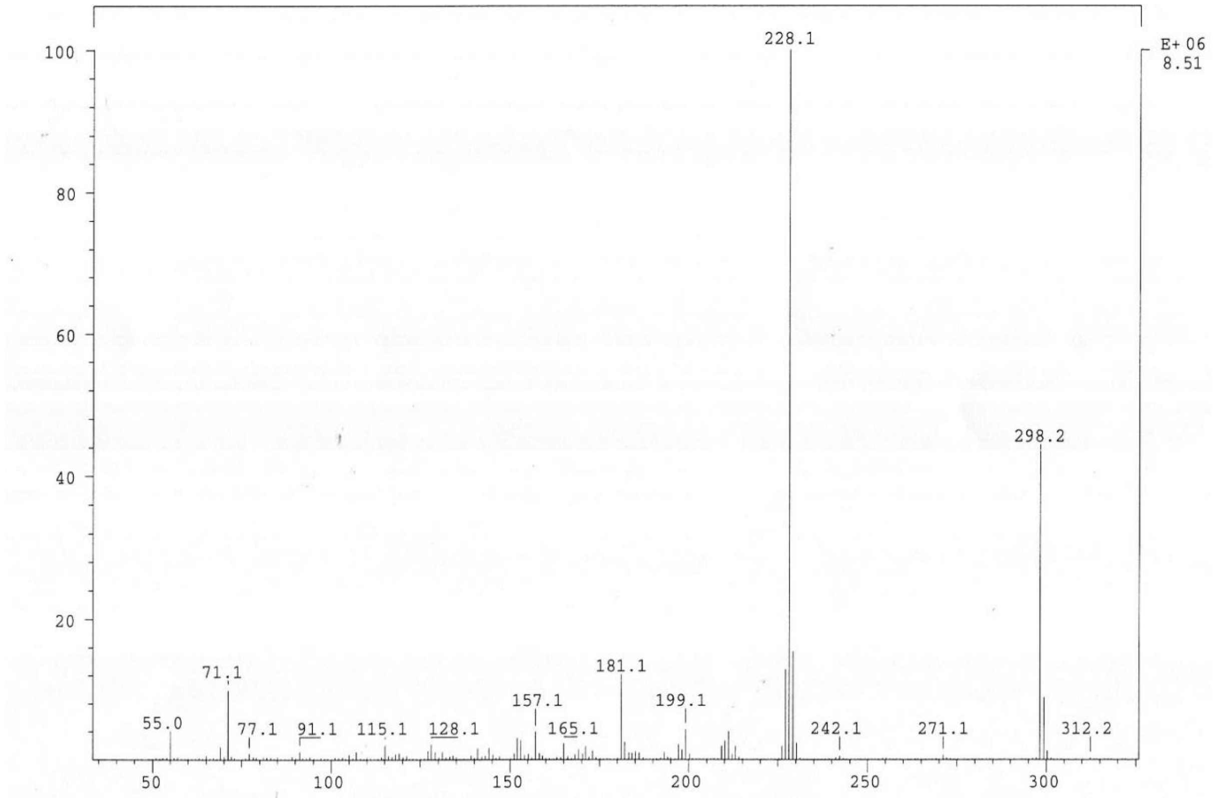

Figure S5: Mass Spectrum of compound **2** (EI-MS Spectrum)

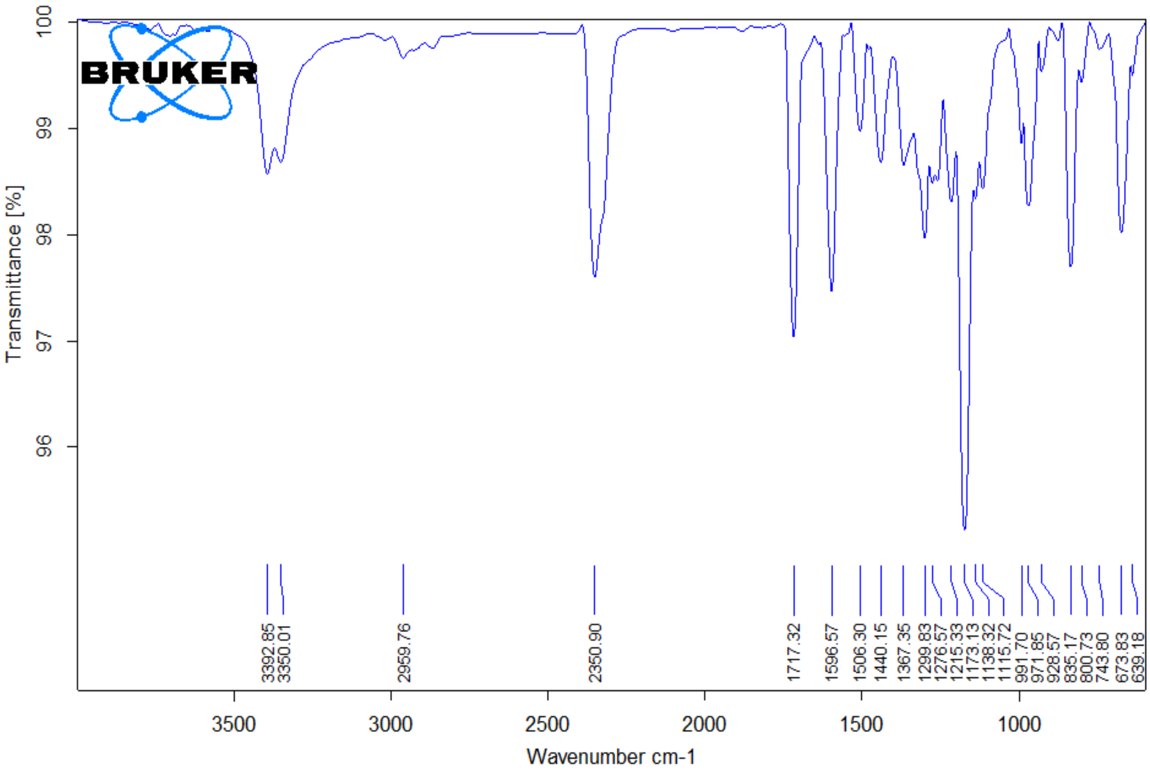

Figure S6: IR spectrum of compound **2**

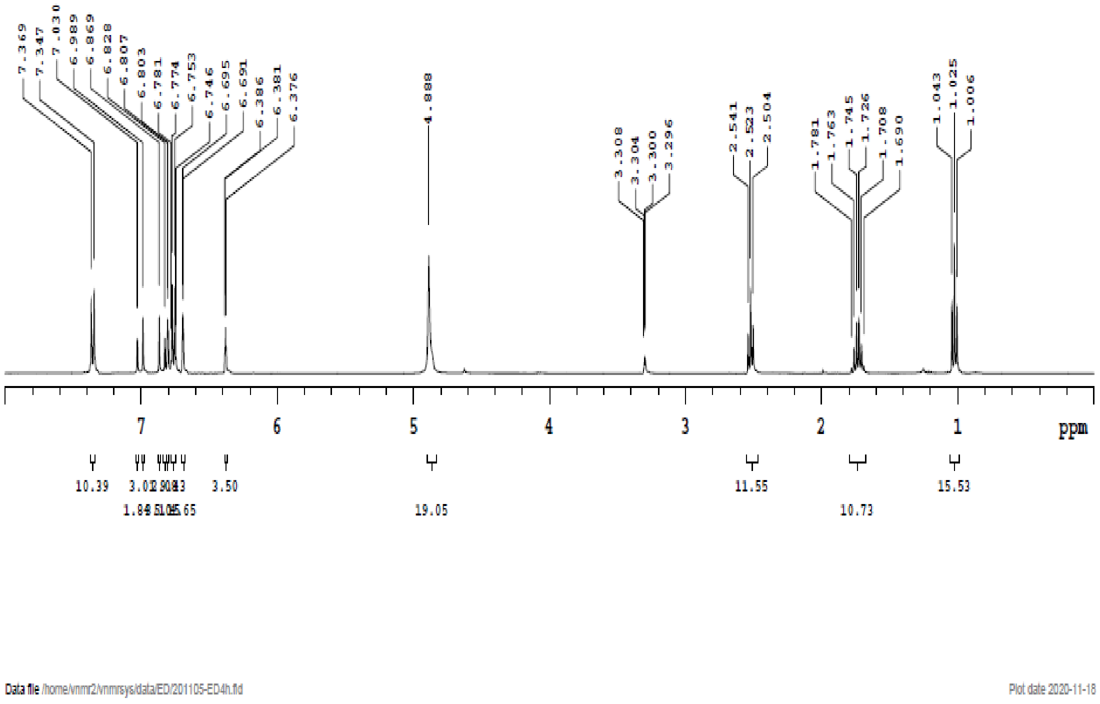

Figure S7: ^{1}H-NMR spectrum of compound **2** in CD_3OD

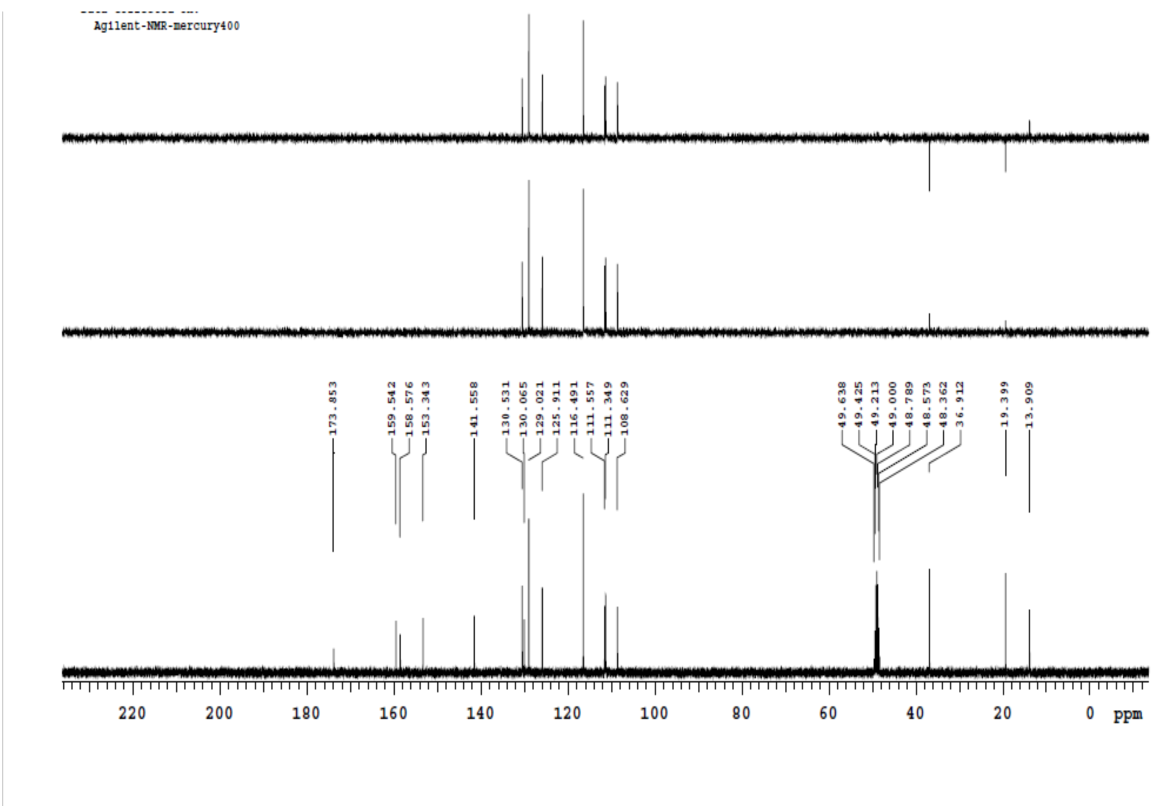

Figure S8: 13C-NMR spectrum and DEPT of compound **2** in CD3OD

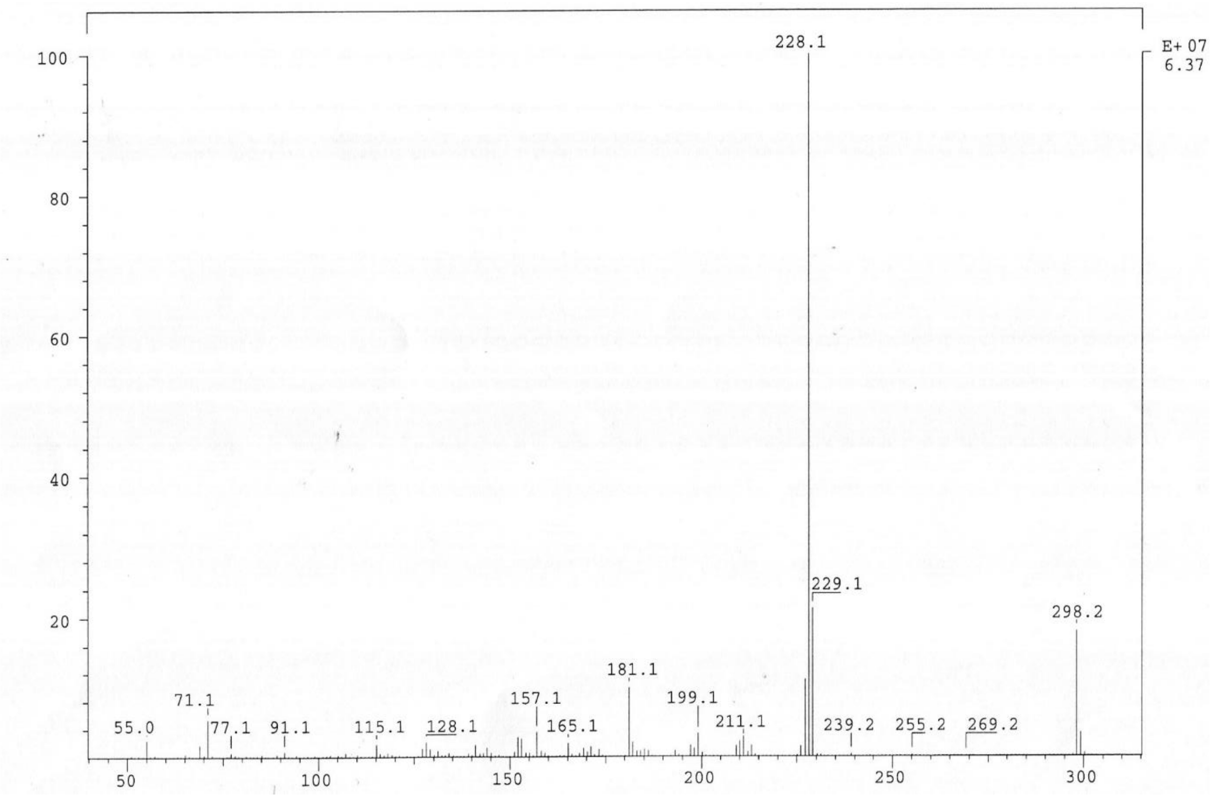

Figure S9: Mass Spectrum of compound **3** (EI-MS Spectrum)

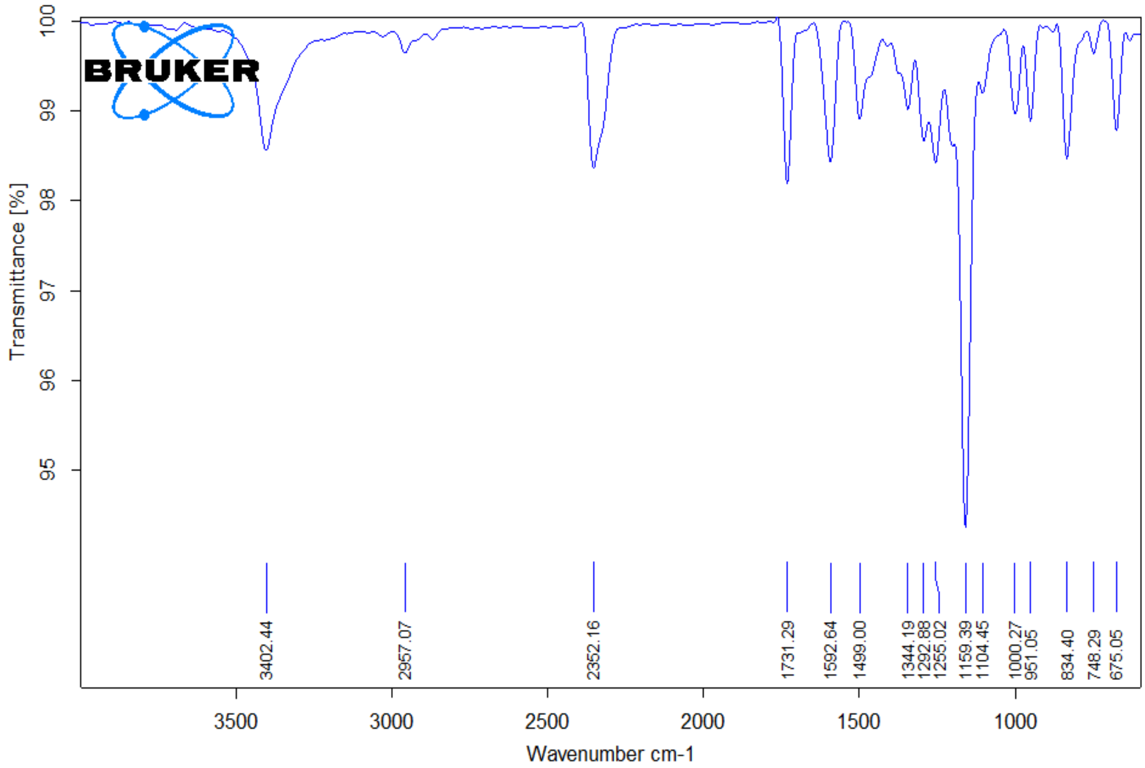

Figure S10: IR spectrum of compound **3**

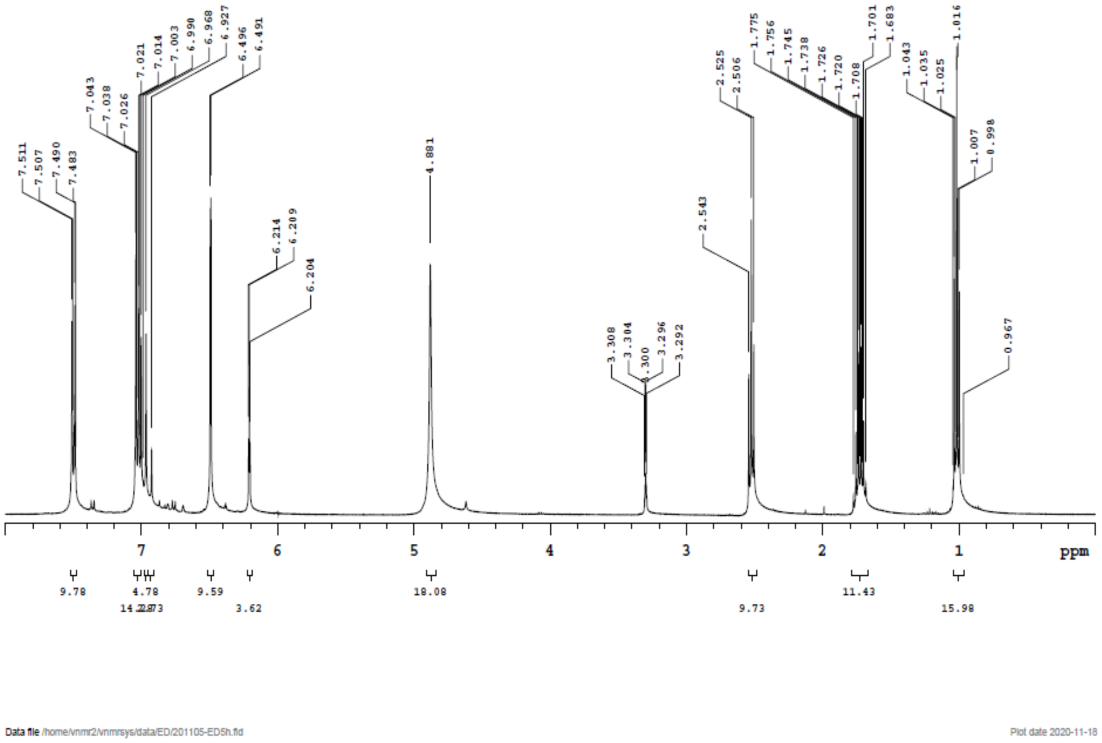

Figure S11: ^{1}H-NMR spectrum of compound **3** in CD_3OD

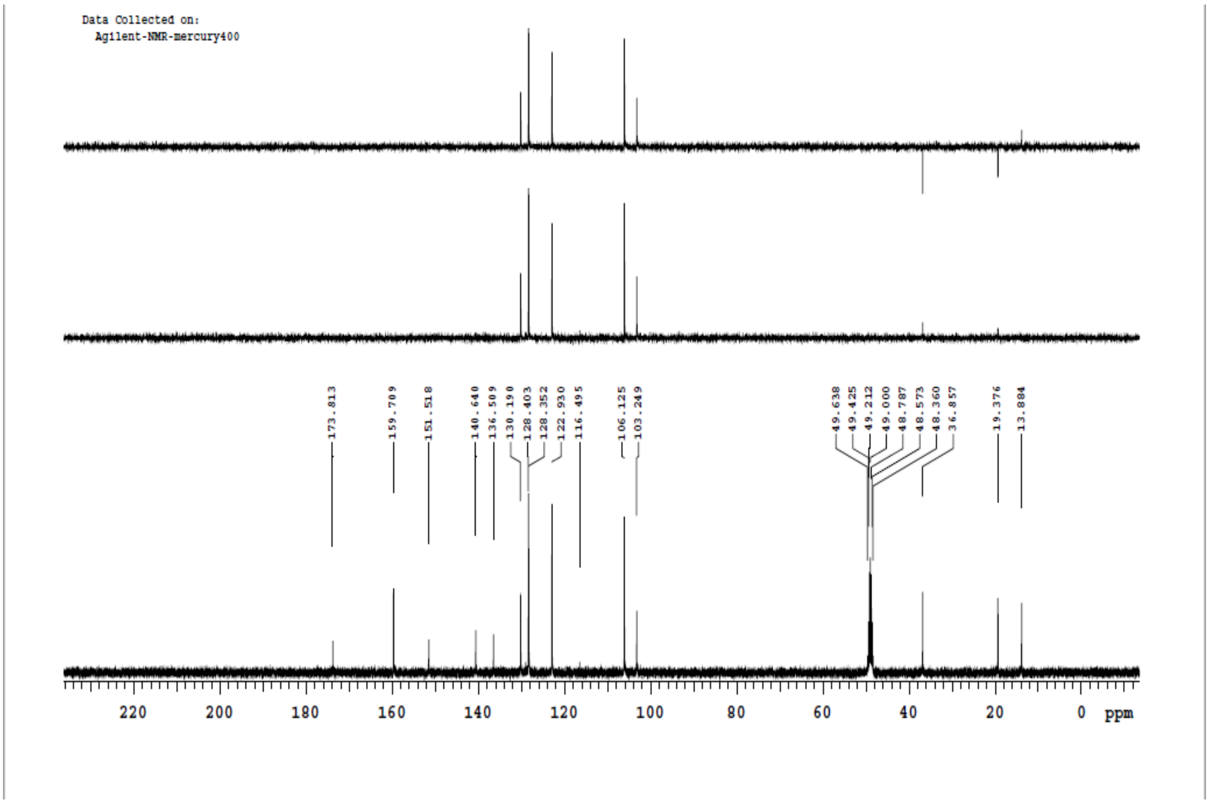

Figure S12: ^{13}C-NMR spectrum and DEPT of compound **3** in CD_3OD

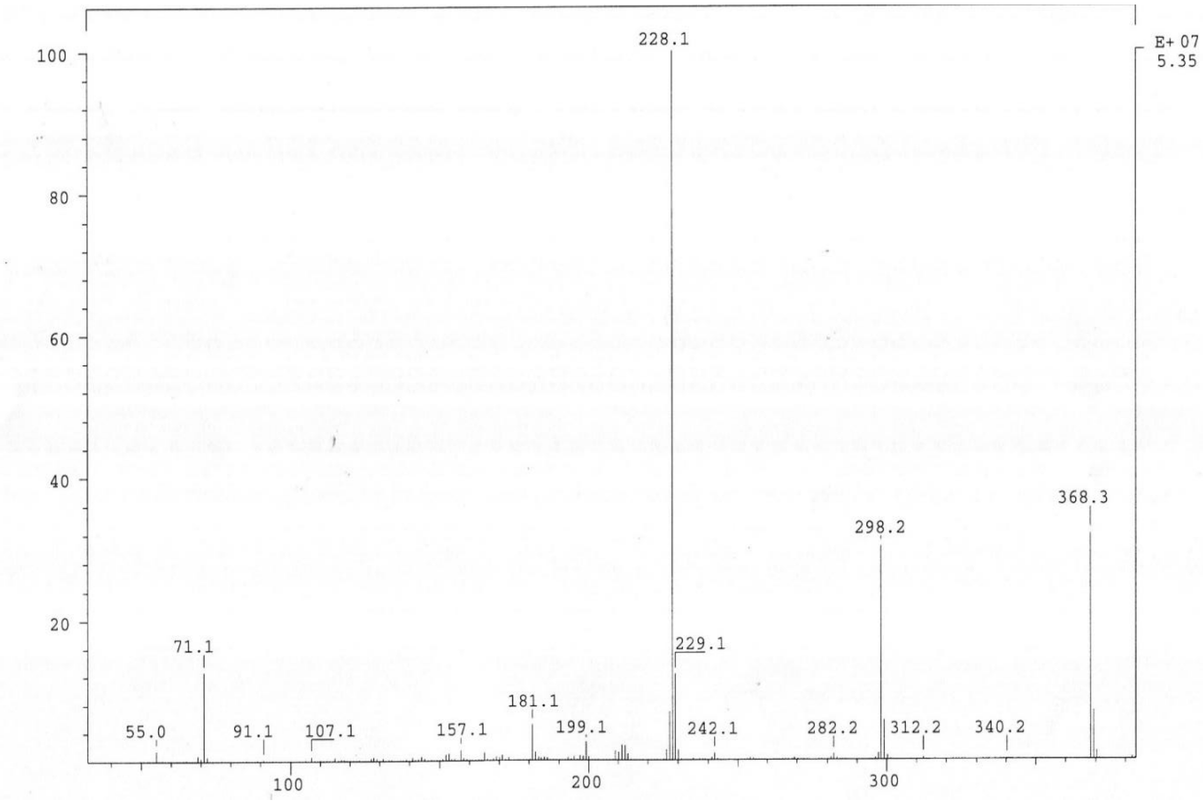

Figure S13: Mass Spectrum of compound **4** (EI-MS Spectrum)

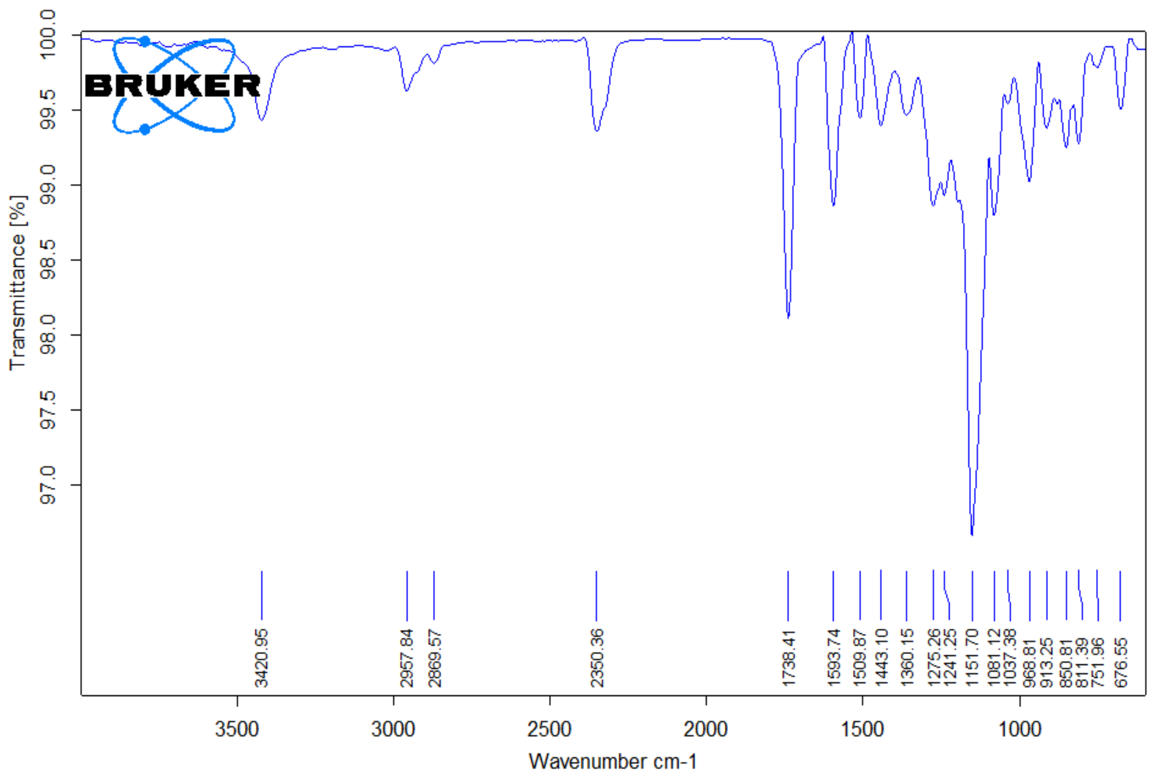

Figure S14: IR spectrum of compound **4**

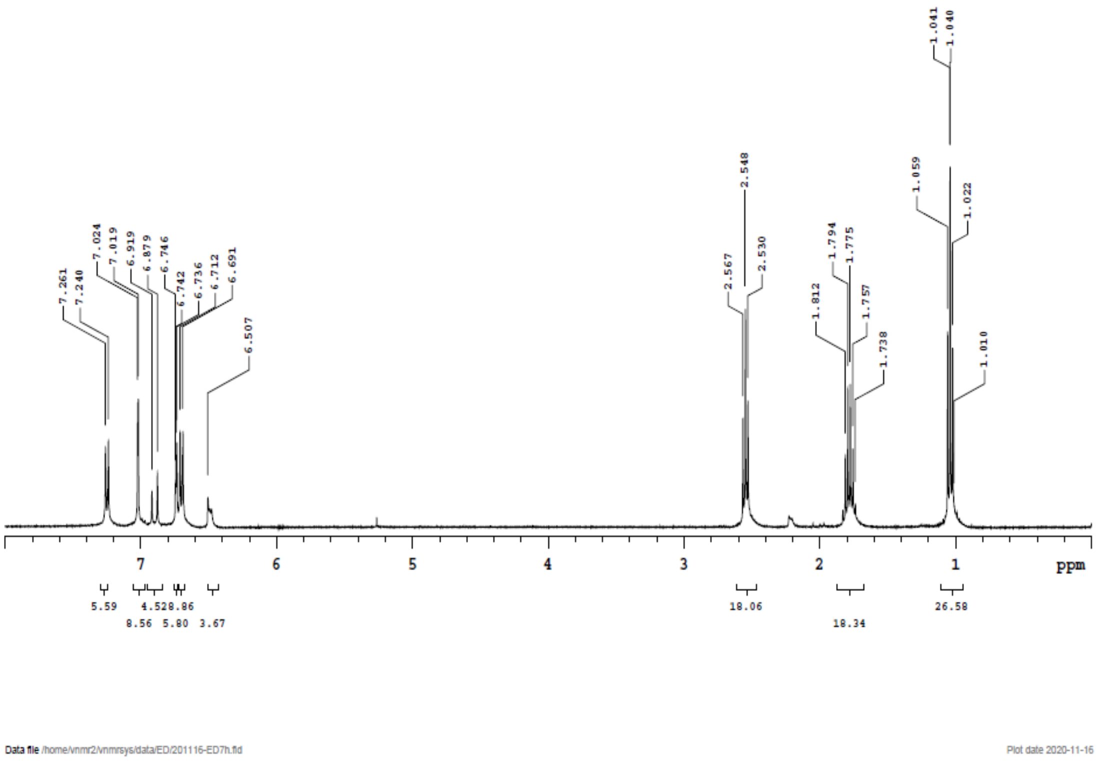

Figure S15: ^{1}H-NMR spectrum of compound **4** in $CDCl_3$

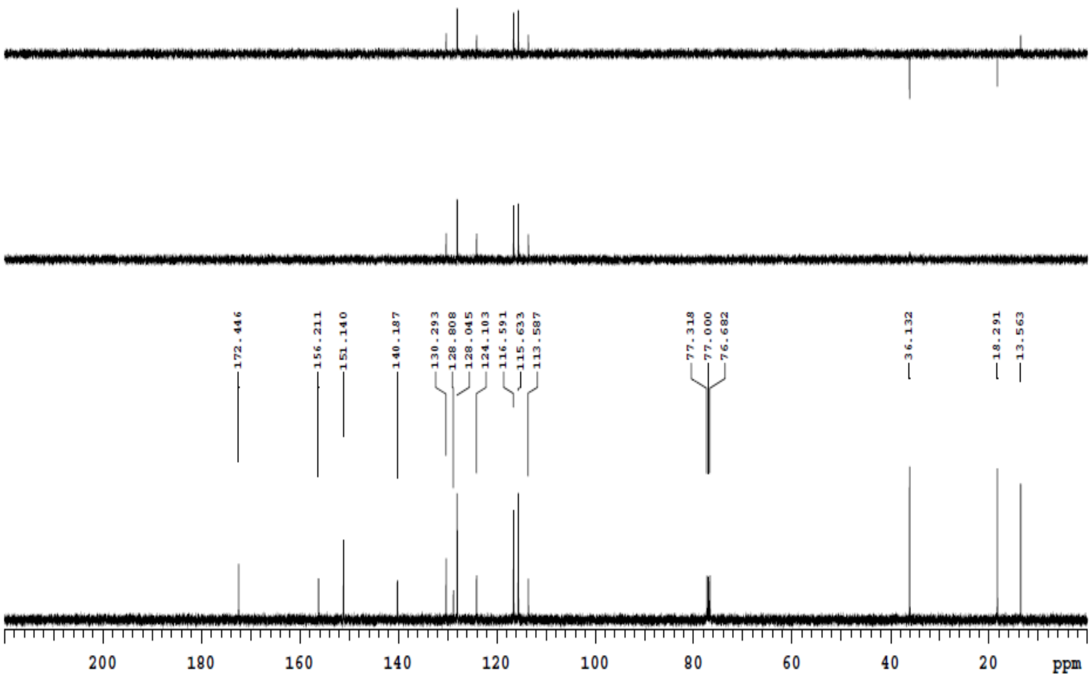

Figure S16: ^{13}C-NMR spectrum and DEPT of compound **4** in $CDCl_3$

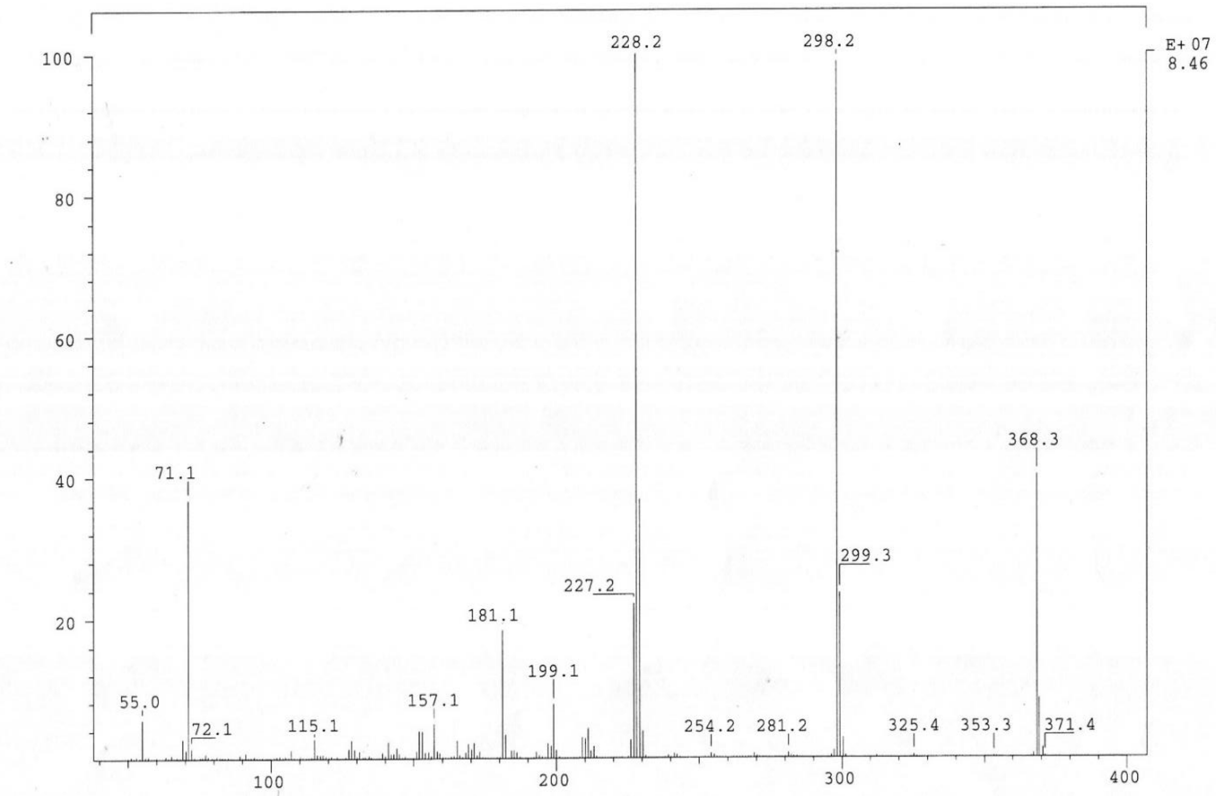

Figure S17: Mass Spectrum of compound **5** (EI-MS Spectrum)

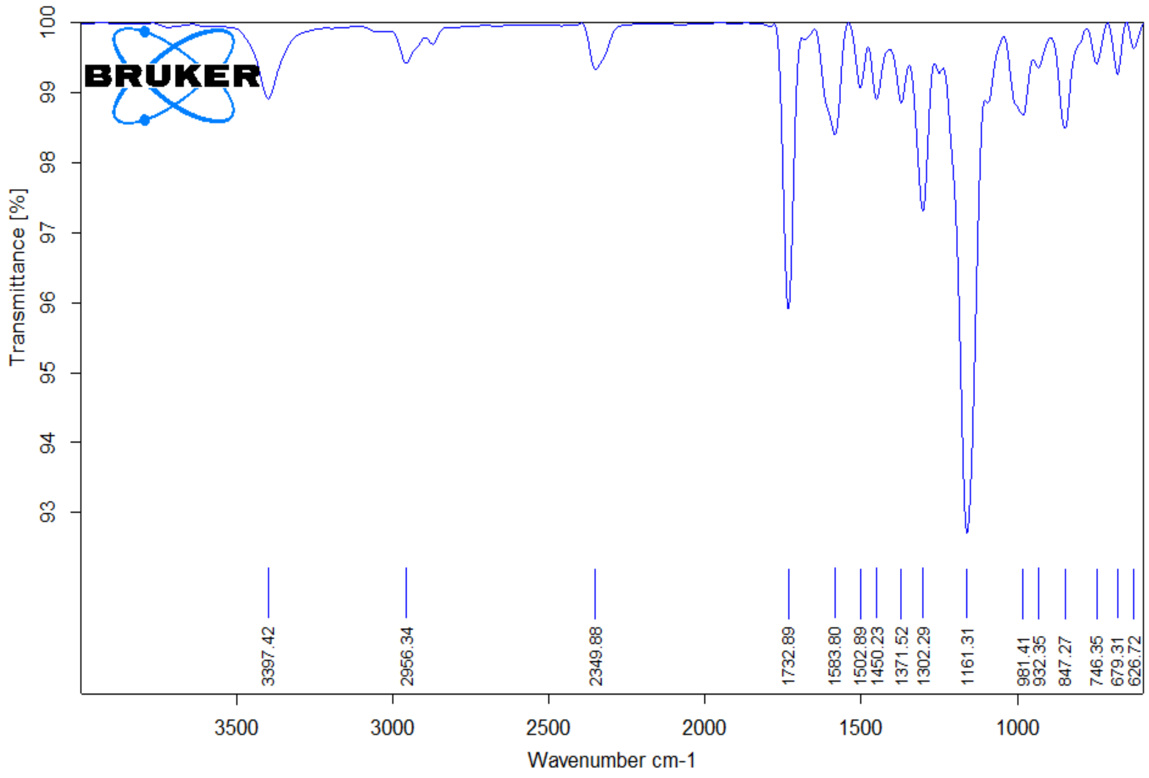

Figure S18: IR spectrum of compound **5**

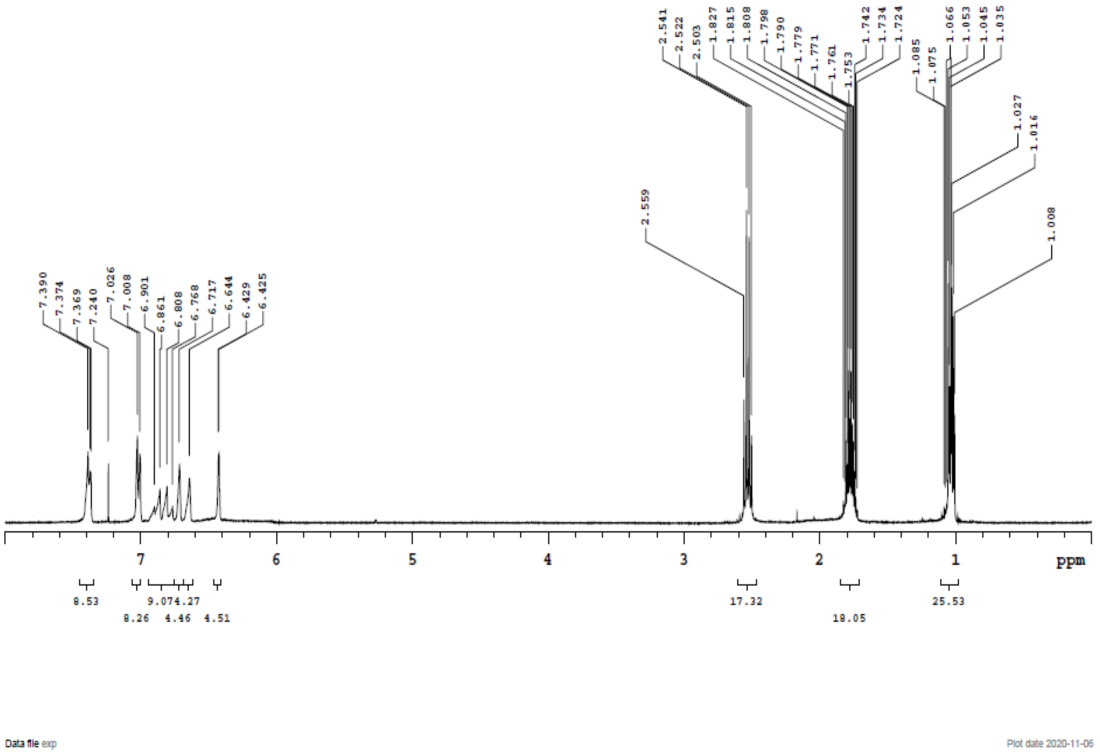

Figure S19: ^{1}H-NMR spectrum of compound **5** in $CDCl_3$

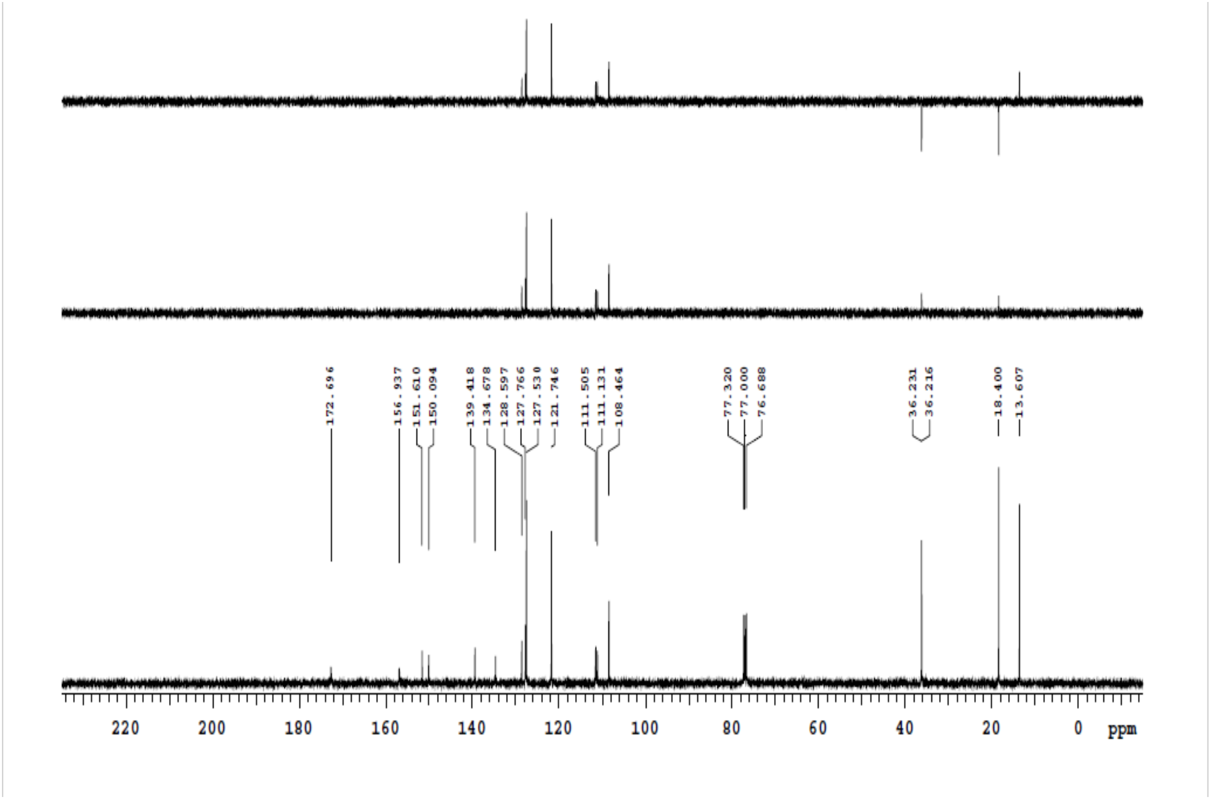

Figure S20: ^{13}C-NMR spectrum and DEPT of compound **5** in $CDCl_3$

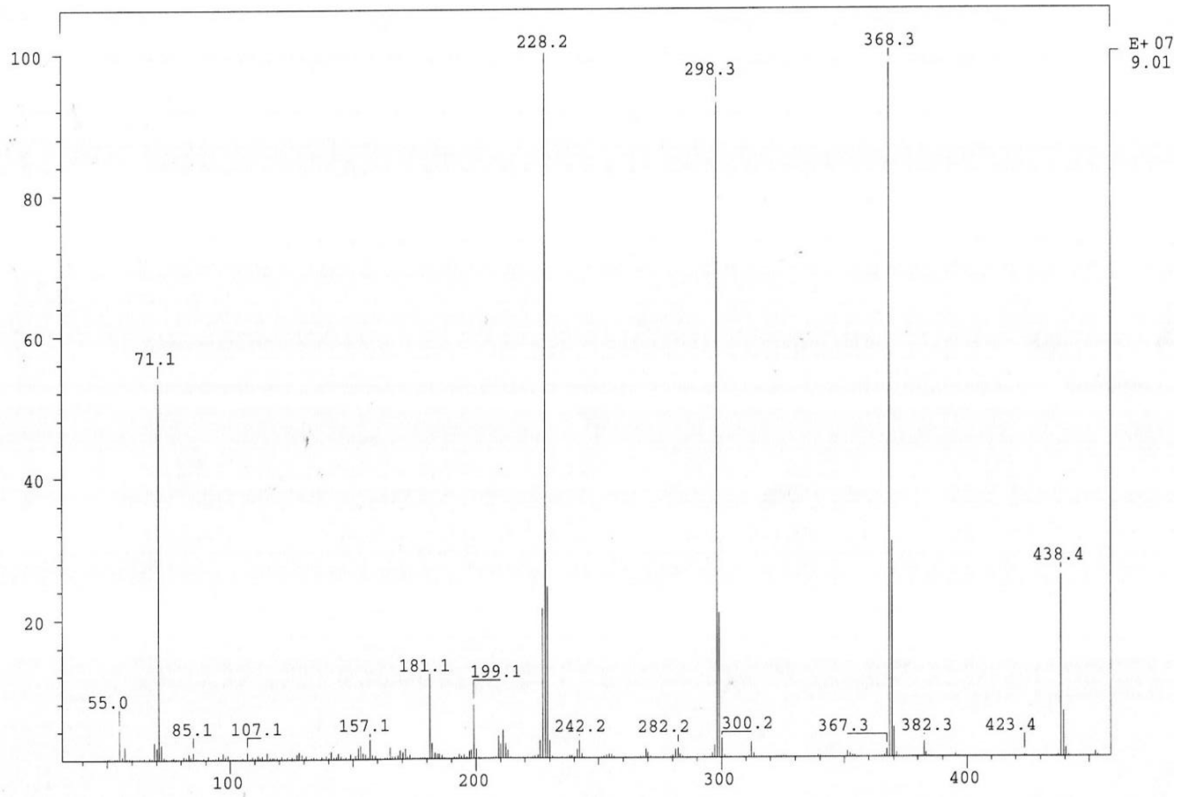

Figure S21: Mass Spectrum of compound **6** (EI-MS Spectrum)

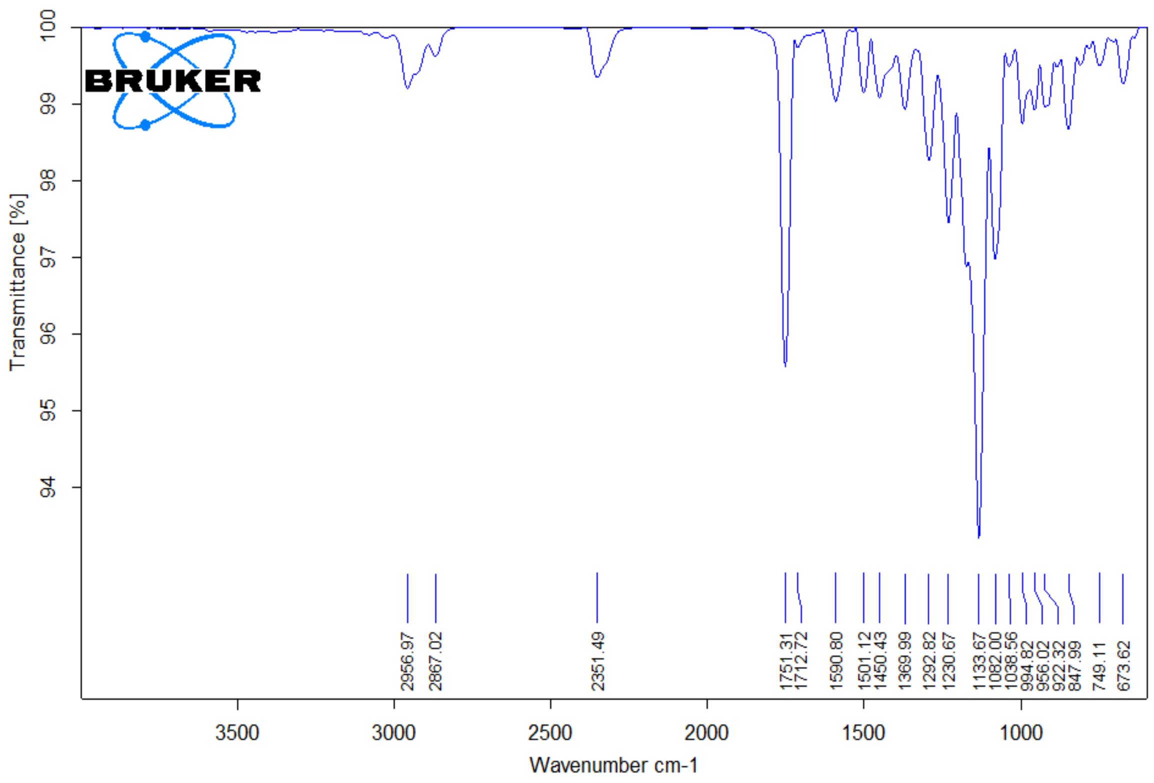

Figure S22: IR spectrum of compound **6**

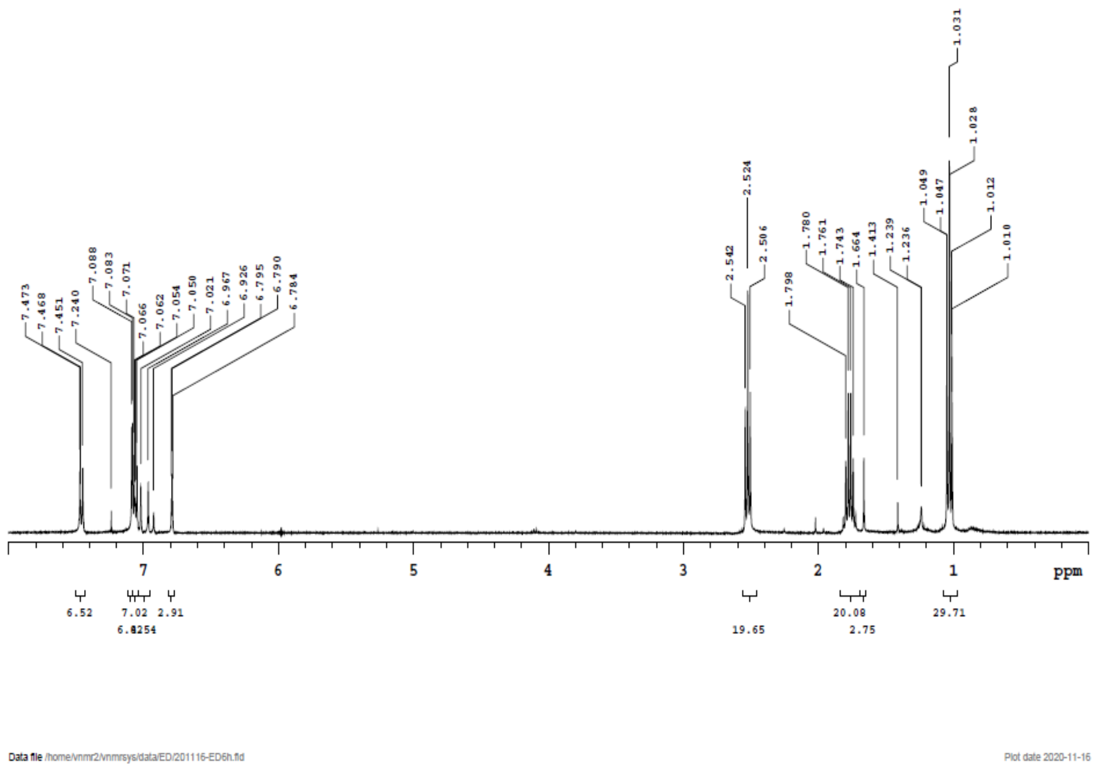

Figure S23: ^{1}H-NMR spectrum of compound **6** in $CDCl_3$

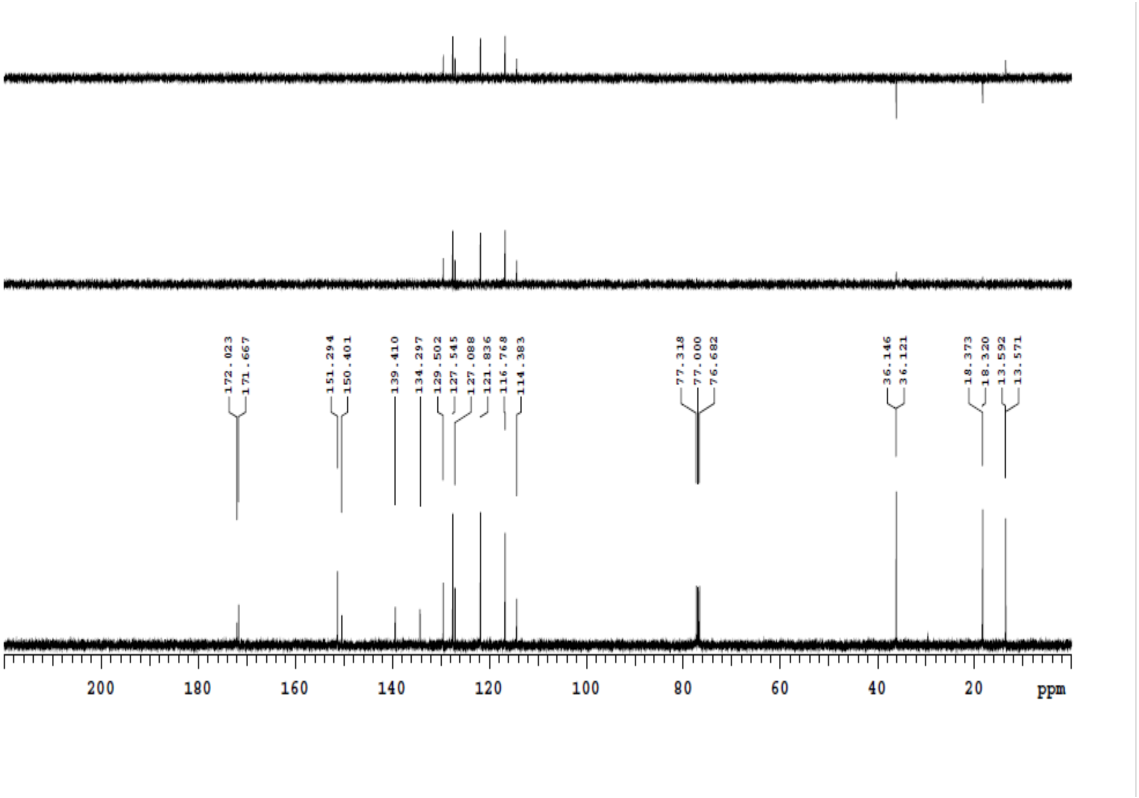

Figure S24: ^{13}C-NMR spectrum and DEPT of compound **6** in $CDCl_3$
